# A Practical Treatise on the Surgical Diseases of the Genito-Urinary Organs, Including Syphilis

**Published:** 1874-07-01

**Authors:** 


### Book Reviews.

*From Hadley Bros.*, 136 *State St.*, *Chicago.*

A PRACTICAL TREATISE ON THE SURGICAL DISEASES OF THE GENITO-URINARY ORGANS, INCLUDING SYPHILIS. Designed as a manual for students and practitioners, with engravings and cases. By W. H. Van Buren, A.M., M.D., Professor of the Principles of Surgery, with Diseases of the Genito-Urinary System and Clinical Surgery, in Bellevue Hospital Medical College, etc., and E. L. Keyes, A.M., M.D., Professor of Dermatology, in Bellevue Hospital Medical College, etc.; pp. 672. New York: D. Appleton & Co., 1874.

Dr. Wood, of Philadelphia, in reviewing Foye's work on the Vapor-bath Treatment of Syphilis, declares that "it looks like the latest volume of poetry, prepared for my lady's boudoir: yet the text is redolent of the charnel-house of vice." But all the world knows that men view different objects from different points of vision. We, for example, prefer to look with Sir Thomas Browne upon "vices and vicious objects with hyperbolical eyes, and rather enlarge their dimensions, that their unseen deformities may not escape sense, and their poisonous parts and stings may appear massy and monstrous; for it is the undiscerned particles and atoms of matter that deceive us. The greatest balsams do lie enveloped in the bodies of the most powerful corrosives, and poisons contain within themselves their own antidote." Bumstead remarks, with equal philosophy, that "it is fortunate, both for the physician and patient, that he whose duty it is to treat the sad consequences of vice, can yet find interest and pleasure in his occupation."

We have premised these observations in a brief notice of the volume before us, because its careful perusal has given us genuine satisfaction. It is a most complete digest of what has long been known, and of what has been more recently discovered in the field of syphilitic and genito-urinary disorders. And the subjects are presented in the most conscientious manner, in unexceptionable style, and with reference to instructive and illustrative cases. It not only embodies the joint experience of its authors, but it contains ample evidence of a critical and thorough research of standard authorities. It is also eminently practical; and while, of course, it is impossible to compress in such a compass, as full a discussion of special subjects as one expects in a monograph, yet enough is said on every point to render the work replete with information, invaluable alike to the student and practitioner. It is, perhaps, not an exaggeration, to say that no single work, upon the same subject, has yet appeared, in this or any foreign language, which is superior to it. And yet, that is no more than we have a right to expect, seeing that it is the latest.

The recent correspondence between Drs. VanBuren and Gouley, on the question of priority in the use of tunneled instruments, has been, doubtless, observed by many whose opinions were undetermined regarding the merits of the controversy. Such, certainly, cannot fail to have a heightened regard for the first-named of the two gentlemen, after reading the very modest note which the author appends to page 127. It is neither aggressive nor violent. It represents the circumstances under which the instruments in question were first devised and employed, in that straight-forward manner, which he who is conscious of his rights might be expected to assume. It reflects in nowise upon the other claimant for the honor, but Dr. Gouley is duly accredited with the improvements made by him, and his instruments are recommended, and called by his name. There can be no question, hereafter, in the profession regarding, at least, the temper of one party to the controversy.

There is a curious defect in both parts of the work, which, since no allusion is made to it by either author, we are entirely at a loss to explain. The subject of syphilis and genito-urinary diseases in women, is almost completely ignored. It may be urged that gynecologists have completely and ably elucidated this branch of the study; but how, we inquire, can one write exhaustively upon gonorrhœa, for example, without devoting a chapter to the disease and its treatment in female patients? Or, how can an author describe syphilitic chancre, and not record the observations of Fournier, Le Blond, Devasse, and others upon the chancres of the os tincæ, mucous patches of the vulva, and the diagnostic differences between simple and specific ulcerations of the cervix?

The forms of syphilitic chancre here described, are the erosive, the ulcerative, the deeply-ulcerative (Hunterian), and the papular; while the table which summarizes the broad, classical characteristics of venereal sores, herpes and balanitic abrasions, (occupying no less than three pages), is the most complete and satisfactory that has yet been compiled.

It is incredible that men of any pretension to accuracy of observation and learning, should hereafter err in the direction which has produced such disastrous results in practice. The primary lesion of syphilis has not the determined character of the primary lesion of vaccinia, or of the disease caused by the malignant pustule. It is not of fixed and definite form. It presents no distinct agreement with any type. The polymorphism of syphilides assuredly belongs to the lesion which precedes the syphilides. In this respect, certainly, they exhibit a striking similarity.

The great and important question for the surgeon, when confronted with a suspicious sore, is *not*, "Have I here an ulcer which corresponds, in its objective features, to this or that recognized type of disease?" Far from it. The questions should be: "Can the syphilitic virus, which is deeply stamped with the triple impress of incubation, non-auto inoculability, and subsequent production of induration, alone and of itself produce this lesion? Has it been implanted upon this previously existing rent, papule, pustule, balanitic abrasion or herpetic vesicle, and, in consequence thereof, modified the evolution of other morbid processes? Has it, though already grafted upon these multiform lesions, not yet produced its own fixed results, which shall yet declare themselves, after a given interval?" Or, to take a different case, "Has this individual, who presents himself to me one week after a suspicious coitus, with no obvious disorder of the mucous surface of his genitals, been yet infected with that virus which may produce a chancre next week, or the week after, and am I justified in promising him, at this time, immunity from disease?"

These are questions of tremendous importance—questions which should be distinctly before the mind of every one who ventures to treat venereal disease. We could wish that the suggestions which they contain were set forth as forcibly in the volume before us as their gravity warrants. The facts are here, but the inferences are left to the judgment and acumen of the reader. If due weight were invariably given to such distinctions, the mentally-tortured practitioner would be in receipt of no such letters as that read by M. P. Diday, in 1872, before the Lyons Medical Society, which has now become historical.

In illustration of one of the points to which reference has been made, Dubuc's cases may be cited, recently published in the *Annales de Dermatologie et de Syphiligraphie.* The lesions, which this author has observed, he terms multiple herpetiform syphilitic chancres, and he describes them as being primarily mere desquamative erosions, varying in number from seven to fourteen, which subsequently become either ulcerative or prominent, so as to resemble muco-papules. They differ from the herpetic lesions in depth of color, tendency to hæmorrhage, and their inaptitude to assume the phases of cicatrization. The epiphenomena of chancres are grafted upon these symptoms.

Whether these be instances of localized disorders, occurring in what Hardy terms the "dartrous diathesis," which become subsequently infected with the syphilitic virus, or whether they constitute a distinct resultant of inoculation, the lesson they teach is an important one. It cannot be too forcibly enunciated, that the circulation of effete products in the blood may produce, in the gouty or rheumatic adult, an eruption of erythema papulatum; in the delicate skin of the infant, strophulus; urticaria, in the nerve paresis of the hysterical woman; squamæ and rhagadæ in the dense epithelium of the palm and sole. So precisely the virus of syphilis upon an exposed and shelterless glans penis; upon the sensitive and habitually closed cul-de-sac of a prepuce; upon the lax lining membrane of the vagina; the horny epithelium of the face; the denser tissue of the os and cervix; upon the broken and the unbroken surface; upon the inflamed and non-inflamed membrane; may, in each and every instance, exhibit different results, which have yet a generic likeness.

The use of the word "lymphitis," in the Treatise of our authors, offends the ear of the etymologist. They acknowledge its critical inaccuracy, but attempt to justify the use of the term on the ground of its general employment, and the fact that it is shorter than "lymphangitis," a substantive which defines itself. We beg leave to demur to the former argument, and to express the opinion that the latter is unworthy of consideration. We prefer the three additional letters, and an inviolate rule of nomenclature and etymology.

On the subject of mercurial inunction, to which additional interest has been lent, since the recent papers of Mr. Jonathan Hutchinson and others, the latest, and, in our opinion the most preferable method, is recommended, that, viz., by the use of the oleates of murcury. The ointment is properly designated, as "more dirty and less efficacious, though it is less expensive." Patients themselves testify on this point, not only in their improved condition, but in the expression of their feelings as to its cleanliness.

We close with an emphatic re-statement of our belief, that for the shelf of the general practitioner, this is the best work on the subject which has yet appeared. It is provided with an exceedingly tasteful form and dress by Messrs. Appleton & Co., and the one hundred and thirty-four cuts are well executed. It is a book in which the profession of America may well take pride, and we congratulate the authors upon their contribution to the general literature of medicine.

